# COVID-19 rapid antigen testing strategies must be evaluated in intended use settings

**DOI:** 10.1016/j.lanwpc.2022.100542

**Published:** 2022-07-13

**Authors:** Jacqueline Dinnes, Clare Davenport

**Affiliations:** Test Evaluation Research Group, Institute of Applied Health Research, University of Birmingham, UK

Testing for severe acute respiratory syndrome coronavirus 2 (SARS-CoV-2) to allow early identification and isolation of those likely to be infectious has been a cornerstone of public health strategies, both to help reduce transmission of infection (via earlier self-isolation or quarantine), and as part of policies to minimize wider societal and economic impacts (allowing faster release from self-isolation or quarantine). The global incidence of COVID-19 has been in relatively steady decline since the beginning of 2022,^1^ however testing strategies remain an important public health tool. Understanding how different tests perform under specific conditions is a pre-requisite for the development of evidence-based testing policies. In healthcare settings, testing strategies aim to minimise missed cases of infection to reduce onward transmission and missed treatment opportunities, while minimising false-positive results that can cause patients to experience unnecessary risk when transferring to dedicated COVID-19 wards.

Rapid antigen tests (RATs) have been a major focus of testing strategies internationally. RATs are less expensive, provide results significantly more quickly, and do not require the same technical expertise or specialist facilities as laboratory-based reverse transcription polymerase chain reaction (RT-PCR), making them attractive for wide scale deployment.^2^ Nevertheless, RATs are known to be less sensitive than RT-PCR,^3^ on average missing up to 27% of RT-PCR positive cases in populations with signs and symptoms of COVID-19 and as much as 45% when used in asymptomatic populations. Although RATs perform better in populations with higher viral loads (usually those who are earlier in the course of infection), accuracy also varies between test kits from different manufacturers, by time from onset of infection or symptoms, and may also be influenced by differences in sample type used, storage and the adequacy of sampling technique and test interpretation.^3^ The impact of a testing strategy is not only driven by the accuracy of the test used but also by the prevalence and the spectrum of infection within that population:^4^^,^^5^ for example, the relative proportions of individuals who are asymptomatic and symptomatic and who have or do not have epidemiological risk factors such as close contact with a COVID-19 case. It is crucial therefore that tests are evaluated in specific use case scenarios prior to full-scale deployment.

In *The Lancet Regional Health − Western Pacific*, Bond and colleagues report a large field-based validation study assessing the accuracy and impact of the Abbott Panbio™ RAT in a hospital emergency department setting during a period of relatively high community transmission of SARS-CoV-2.^6^ A standardised triage system for emergency admissions based on prior COVID-19 test results, the presence of signs and symptoms of COVID-19 or of likely epidemiological exposure to infection was already established at the hospital, with safety measures such as patient isolation and level of staff PPE guided by RT-PCR results on admission. The observed sensitivity of the RAT in this setting was 75.5% overall. Grouping by risk category (five categories from high to no risk) suggested a clear effect from the presence of symptoms (sensitivity 45 percentage points higher in symptomatic compared to asymptomatic individuals) but also, to a lesser extent, from epidemiological exposure (sensitivity 81.7% with both symptoms and epidemiological exposure compared to 75.4% with symptoms only). Almost half of cases missed by the RAT were within the first week of symptoms, or had viral loads in the higher range, demonstrating that the RAT strategy could not be used to downscale safety measures in those who test negative.

The lack of observed false-positive results (specificity 100%^6^) in a large study such as this is surprising given that we know that RAT tests do have a very small risk of misclassification of disease negative individuals (average specificity 99.6%^3^). When infection rates are high or RATs are used in those at higher risk of infection, the potential harm from false positive results is likely outweighed by earlier detection of true cases. However, this may not be the case in lower risk groups where prevalence of infection is lower. Importantly in this study, the impact of introducing the RAT on length of stay in the emergency department (prior to transfer to an inpatient ward) was reduced for RAT positive patients in High or At risk groups compared to those in the same group who were RAT negative (274 minutes compared to 421 minutes). No difference in length of stay was observed according to RAT result for low or no risk patients.

By expediating care pathway decisions, implementation of the RAT testing policy in the study setting potentially reduced onward transmission of infection in almost a third of PCR positive cases (i.e., the RAT positive higher risk group). RAT testing was not implemented in all those presenting to the emergency department during the course of the study however, with those at lower risk of infection less likely to be tested. As a result, the overall observed accuracy and potential impact from reduced length of stay in those at higher risk may not be reproducible if RAT testing was to be used consistently across risk groups as part of hospital admission triage policy. The pre-existence of standardised symptom triage and the level of staff training in triage and in RAT test use and interpretation, are additional factors that will affect the transferability of these results to other settings

Bond and colleagues^6^ have demonstrated that RAT testing may be a useful strategy when used in combination with COVID-19 symptom and epidemiological profiles, particularly during a period of rising infection rates and when implemented as triage to RT-PCR. Notably, the study also highlights the importance of validating tests in the specific context in which they will be used and of evaluation of the potential impact from tests beyond test accuracy alone.

## Contributors

J.D. and C.D. were responsible for the writing of the commentary.

## Declaration of interests

The authors declare no conflict of interest.
